# Prevalence and Associated Risk Factors of Insomnia Among Adults in Riyadh, Saudi Arabia

**DOI:** 10.7759/cureus.67086

**Published:** 2024-08-17

**Authors:** Hareth Almajid, Abdulrahman M Elnasieh, Alaa A Alnamlah

**Affiliations:** 1 Family Medicine, King Saud Medical City, Riyadh, SAU

**Keywords:** saudi arabia, risk factors, sleep quality, prevalence, insomnia

## Abstract

Background

Insomnia is a common sleep disorder with significant effects on physical and mental health. This study aimed to document the prevalence of insomnia and its associated risk factors among adults in Riyadh, Saudi Arabia.

Materials and methods

A cross-sectional study was conducted in Riyadh using the Sleep Condition Indicator Questionnaire (SCIQ). Data were collected from 548 participants. The inclusion criteria encompassed adults aged 18 and above living in Riyadh, Saudi Arabia. Information on sociodemographic characteristics, medical history, and sleep patterns was gathered through a translated eight-item SCIQ (score ≤16=insomnia). Statistical analysis involved descriptive and inferential statistics using IBM SPSS Statistics for Windows, Version 27.0 (Released 2020; IBM Corp., Armonk, New York, United States).

Results

The findings revealed a significant prevalence of insomnia, affecting 219 individuals (40%). Insomnia was significantly associated with the Diagnostic and Statistical Manual of Mental Disorders, Fifth Edition (DSM-5) criteria, including difficulty initiating or maintaining sleep, frequency of sleep disturbances, significant distress, and duration of sleep disturbances (p<0.001 for each). The highest prevalence of insomnia was observed in the 40-60-year age group (n=91, 45.7%, p=0.014), among smokers (n=27, 60%, p=0.0063), and among obese individuals (n=20, 54.1%, p=0.035). Additionally, insomnia was strongly associated with anxiety (n=49, 66.2%, p<0.001), depression (n=27, 54%, p=0.033), and hypertension (n=24, 58.5%, p=0.011).

Conclusion

This study highlighted the high prevalence of insomnia among adults in Riyadh, particularly among older adults, smokers, and those with chronic health conditions. These findings underscore the need for targeted interventions to address insomnia and its associated risk factors. Future research should focus on longitudinal studies to establish causal relationships and explore the impacts of lifestyle and genetic factors on insomnia.

## Introduction

Sleep is a naturally recurring state of mind and body, characterized by altered consciousness, relatively inhibited sensory activity, inhibition of nearly all voluntary muscles, and reduced interactions with surroundings. Indeed, our health and well-being are profoundly connected to our sleep. Sleep is fundamental to a healthy individual, and without the right amount of it, we cannot function properly, increasing the risk of various health issues. Numerous chronic medical conditions such as hypertension, diabetes mellitus, and coronary artery disease are associated with sleep disorders [1].

Sleep enhances immune function, promotes glucose metabolism, lowers the risk of developing diabetes, and increases alertness and activity during the day [2]. Insufficient sleep can impair alertness, attention, and cognitive processes [3]. Insomnia, the most prevalent sleep disturbance, affects millions of people as either a primary or a coexisting condition [4]. It impacts nearly a third of the population, with a prevalence ranging from 10% to 33% worldwide [5,6]. Insomnia is characterized by dissatisfaction with the quantity or quality of sleep, leading to significant discomfort and deficits in daily functioning. Common symptoms include difficulties falling asleep, trouble staying asleep, and early morning awakenings with an inability to return to sleep [7]. Previously considered secondary to anxiety or depression, insomnia is now recognized as a major issue requiring focused treatment regardless of other comorbidities [5].

Studies on the prevalence of insomnia in adults are critical due to its substantial impact on physical and mental health and overall quality of life. Globally, the prevalence of insomnia and its associated risk factors have been extensively studied, particularly in the Western world. For instance, a study in Swiss primary care reported an insomnia prevalence of 31%, with 11% meeting the Diagnostic and Statistical Manual of Mental Disorders, Fifth Edition (DSM-5) criteria for chronic insomnia. Additionally, 49% and 38% of those with chronic insomnia also had depression and anxiety, respectively [6]. In Turkey, a study among the older population (60 years or older) found a prevalence of insomnia at 51% [4].

In the Arabian Gulf region, studies have focused on specific groups and ages, such as students, adolescents, primary care center patients, health workers, and patients with chronic diseases like end-stage renal disease [7-9]. In Qatar, a study measured the prevalence of insomnia at 5.5% using the DSM-5 criteria, with 2% of the sample testing positive for depression and 3.4% for anxiety in the previous two weeks [7]. In Saudi Arabia, the prevalence of insomnia and its associated risk factors have not been extensively studied, despite the serious impact of insomnia on physical and mental health. A research study in the primary healthcare facilities of the Aseer region reported a 60% prevalence of insomnia, particularly higher among individuals aged 25-40 and those with low income [5]. Another study reported an insomnia prevalence of 54.4% using the Athens Sleep Questionnaire (ASQ) [10].

Due to the scarcity of studies measuring the prevalence of insomnia in Saudi Arabia, as well as its effects on mental health and associated risks, this study aims to determine the prevalence of insomnia among adults in Riyadh, Saudi Arabia, and to identify key associated risk factors, including sociodemographic and health-related factors. The findings will help guide targeted public health interventions.

## Materials and methods

Study design

This cross-sectional study was conducted in Riyadh, Saudi Arabia. Based on an assumed response distribution of 50%, a confidence interval of 95%, and a 5% margin of error, the calculated sample size was 385 using the Raosoft software. However, to enhance the reliability and generalizability of the findings, the sample size was increased to 548 participants.

Study population

The study included 548 adults from Riyadh aged 18 years and older, with no upper age limit, to ensure that it examines sleep patterns and behaviors across the entire adult lifespan. Individuals younger than 18 years and those who did not complete the questionnaire were excluded. The increase in sample size beyond the initially calculated 385 participants was intended to ensure a more robust data set, account for potential non-responses, and improve the study's overall validity and statistical power.

Questionnaire and data collection

Data were collected using the eight-item Sleep Condition Indicator (SCI), based on the DSM-5 criteria. The questionnaire was distributed to participants in Riyadh after obtaining their consent. An online questionnaire was created using Google Forms and disseminated through social media platforms such as WhatsApp and Telegram. An Arabic version of the questionnaire was available for participants to complete. A pilot sample of 15-20 participants was initially used to test the applicability of the questionnaire, and these participants were included in the final sample.

Data variables

The study measured both dependent and independent variables. The dependent variable was the disorder being studied, namely, insomnia (the outcome). The independent variables included presumed associated risk factors, which could influence the outcome. These predictors included the anthropometric characteristics of the participants and associated comorbidities.

By increasing the sample size from the initially calculated 385 to 548, the study aimed to enhance the precision of its estimates and ensure a more comprehensive understanding of the prevalence and associated risk factors of insomnia among adults in Riyadh.

Ethical considerations

The study was approved by the Research and Innovation Center of King Saud Medical City (KSMC) Riyadh Institutional Review Board (approval number: H1RI-13-Sep23-04). Informed consent was obtained from all participants after explaining the research objectives. The study participants remained anonymous throughout the entire research process. Participation in this research was entirely voluntary. The investigators of this research had no conflicts of interest to declare.

Statistical analysis plan

The analysis involved both descriptive and inferential statistics. Descriptive statistics were used to summarize and describe the characteristics of the study participants and their responses. Frequencies and percentages were calculated for categorical variables, such as the participants' responses to the Sleep Condition Indicator Questionnaire (SCIQ). Additionally, chi-squared tests and Fisher's exact tests (where the expected count in cells was less than 5) were conducted to compare insomnia among different categorical groups. The significance level for all statistical tests was set at p<0.05, indicating a 95% confidence interval. All statistical calculations were performed using IBM SPSS Statistics for Windows, Version 27.0 (Released 2020; IBM Corp., Armonk, New York, United States). The study included a total of 548 cases.

Insomnia

The eight-item SCI was used to assess the presence and severity of insomnia among study participants. This standardized tool includes eight items that capture various aspects of sleep quality, duration, and the impact of sleep disturbances on daily functioning. Each item on the SCIQ is scored on a scale from 0 to 4. The total score is calculated by summing the scores of all individual items. Those who scored 16 or less were considered to be suffering from insomnia. As operationalized by the SCI, we measured four main criteria for insomnia disorder as per the DSM-5 criteria. The first criterion was difficulty initiating or maintaining sleep, based on reports of taking more than 30 minutes to fall asleep or staying awake for more than 30 minutes in total after waking up during the night, and rating sleep quality as average, poor, or very poor. The second criterion was significant distress, which was determined by endorsing "somewhat," "much," or "very much" for the extent that poor sleep in the past month has troubled the respondent in general and endorsing "somewhat," "much," or "very much" to one of two questions about the extent poor sleep in the past month has affected the respondent's mood, energy, relationships, concentration, productivity, or ability to stay awake. The third criterion involved the frequency of sleep disturbances, requiring the endorsement of a minimum of three nights per week for the frequency of encountering sleep problems. The fourth criterion was related to the duration of sleep disturbances, based on endorsing a minimum duration of three months for having sleep problems.

## Results

Table 1 presents the sociodemographic characteristics of the 548 study participants, offering a detailed overview of the sample. The majority of participants are Saudi nationals, constituting 81.9% (449 individuals), while non-Saudis make up 18.1% (99 individuals). The gender distribution reveals a higher number of females, who comprise 57.3% (314 participants), compared to males at 42.7% (234 participants).

**Table 1:** Sociodemographic characteristics of participants in the study (n=548) N: frequency; %: percentage; * others: encompassing various other conditions

Variable		N (%)
Nationality	Saudi	449 (81.9%)
	Non-Saudi	99 (18.1%)
Gender	Female	314 (57.3%)
	Male	234 (42.7%)
Age (years)	18-24	83 (15.1%)
	25-39	242 (44.2%)
	40-60	199 (36.3%)
	>60	24 (4.4%)
Occupation	Employed	282 (51.5%)
	Unemployed	266 (48.5%)
Marital status	Single	172 (31.4%)
	Married	351 (64.1%)
	Divorced	16 (2.9%)
	Widower	9 (1.6%)
Highest education level	Primary or middle school	12 (2.2%)
	Secondary school	108 (19.7%)
	University	325 (59.3%)
	Postgraduate	101 (18.4%)
	Uneducated	2 (0.4%)
Past medical history	Anxiety	74 (13.7%)
	Depression	50 (9.2%)
	Asthma	40 (7.4%)
	Diabetes	37 (6.8%)
	High blood pressure	41 (7.6%)
	Others*	28 (5.1%)
	None	278 (50.7%)
Smoking status	Yes	45 (8.2%)
	No	503 (91.8%)
Weight (kg)	<40 kg	1 (0.2%)
	40-49 kg	35 (6.4%)
	50-59 kg	83 (15.1%)
	60-69 kg	148 (27%)
	70-79 kg	119 (21.7%)
	80-89 kg	80 (14.6%)
	90-99 kg	45 (8.2%)
	>100 kg	37 (6.8%)
Height (cm)	140-149 cm	17 (3.1%)
	150-159 cm	197 (35.9%)
	160-169 cm	199 (36.3%)
	170-179 cm	98 (17.9%)
	180-189 cm	35 (6.4%)
	190 cm +	2 (0.4%)

Regarding age distribution, the largest group is between 25 and 39 years old, accounting for 44.2% (242 individuals). This is followed by the 40-60-year age group with 36.3% (199 participants), the 18-24-year age group at 15.1% (83 participants), and the smallest group, those aged 60 and above, comprising 4.4% (24 participants).

Occupationally, most participants are employed, representing 51.5% (282 individuals). Marital status data shows that the majority of participants are married, totaling 64.1% (351 individuals). Educational levels indicate that a predominant number of participants hold university degrees, accounting for 59.3% (325 participants), followed by those with secondary school education at 19.7% (108 participants).

In terms of past medical history, anxiety is the most common condition, affecting 13.7% (74 individuals), followed by depression in 9.2% (50 individuals), asthma in 7.3% (40 individuals), diabetes in 6.8% (37 individuals), and high blood pressure in 7.5% (41 individuals). The "Others" category, encompassing various other conditions, affects 5.1% (28 individuals).

Smoking status reveals that a large majority of participants, 91.8% (503 individuals), do not smoke, whereas 8.2% (45 individuals) do.

Regarding weight, the largest group weighs between 60 and 69 kg, with 27% (148 participants). This is followed by those in the 70-79 kg range (21.7%, 119 participants) and the 50-59 kg range (15.1%, 83 participants). Height distribution shows that most participants are between 160-169 cm (36.3%, 199 participants) and 150-159 cm (35.9%, 197 participants). The remaining participants are distributed across other height categories: 140-149 cm (3.1%, 17 participants), 170-179 cm (17.9%, 98 participants), 180-189 cm (6.4%, 35 participants), and 190 cm and above (0.4%, two participants).

Table 2 provides a comprehensive overview of the sleep patterns of the study participants based on their responses to the SCIQ. The time it takes for participants to fall asleep varies, with the majority (37.2%) falling asleep within 16-30 minutes, followed by 25.9% who fall asleep within 0-15 minutes. The mean time to fall asleep is 2.6±1.2 minutes. When asked about the duration of wakefulness during the night, 52% of participants reported being awake for 0-15 minutes. Meanwhile, 22.6% are awake for 16-30 minutes, 10.6% for 31-45 minutes, 8.2% for 46-60 minutes, and 6.6% for more than 61 minutes. The mean wakefulness duration is 3.1±1.2 minutes.

**Table 2:** Response of the participants on the SCIQ Score is given in brackets SCIQ: Sleep Condition Indicator Questionnaire; N: frequency; %: percentage; SD: standard deviation

Item	Response (score)	N (%)	Mean±SD
How long does it take you to fall asleep?	≥61 min (0)	50 (9.1%)	2.6±1.2
	46-60 min (1)	47 (8.6%)	
	31-45 min (2)	105 (19.2%)	
	16-30 min (3)	204 (37.2%)	
	0-15 min (4)	142 (25.9%)	
How much time does it take during your waking period to complete your sleep?	≥61 min (0)	36 (6.6%)	3.1±1.2
	46-60 min (1)	45 (8.2%)	
	31-45 min (2)	58 (10.6%)	
	16-30 min (3)	124 (22.6%)	
	0-15 min (4)	285 (52%)	
How many nights a week do you have trouble sleeping?	5-7 (0)	77 (14.1%)	2.5±1.4
	4 (1)	67 (12.2%)	
	3 (2)	92 (16.8%)	
	2 (3)	126 (23%)	
	0-1 (4)	186 (33.9%)	
How do you rate your sleep quality?	Very poor (0)	14 (2.6%)	2.7±1.1
	Poor (1)	56 (10.2%)	
	Average (2)	176 (32.1%)	
	Good (3)	144 (26.3%)	
	Very good (4)	158 (28.8%)	
Does lack of sleep affect your mood, energy, or relationships?	Very much (0)	59 (10.8%)	1.8±1.0
	Much (1)	153 (27.9%)	
	Somewhat (2)	213 (38.9%)	
	A little (3)	89 (16.2%)	
	Not at all (4)	34 (6.2%)	
Does poor sleep affect your focus, productivity, or ability to stay awake?	Very much (0)	57 (10.4%)	1.7±1.1
	Much (1)	190 (34.7%)	
	Somewhat (2)	164 (29.9%)	
	A little (3)	108 (19.7%)	
	Not at all (4)	29 (5.3%)	
Does bad sleep affect you in general?	Very much (0)	59 (10.8%)	1.8±1.0
	Much (1)	154 (28.1%)	
	Somewhat (2)	200 (36.5%)	
	A little (3)	105 (19.2%)	
	Not at all (4)	30 (5.5%)	
How long have you had trouble sleeping?	More than a year (0)	230 (42%)	1.9±1.8
	7-12 months (1)	25 (4.6%)	
	3-6 months (2)	54 (9.9%)	
	1-2 months (3)	63 (11.5%)	
	I don't have a problem/<1 month (4)	176 (32.1%)	

Regarding sleep quality, 28.8% of participants rated their sleep as very good, 26.3% as good, 32.1% as average, 10.2% as poor, and 2.6% as very poor. The mean sleep quality score is 2.7±1.1. The impact of poor sleep on mood, energy, or relationships was reported as very much by 10.8% of participants, much by 27.9%, somewhat by 38.9%, a little by 16.2%, and not at all by 6.2%. The mean impact score is 1.8±1.0.

Concerning the duration of sleep problems, 42% of participants have experienced sleep problems for more than a year, 4.6% for 7-12 months, 9.9% for 3-6 months, and 11.5% for 1-2 months, and 32.1% reported no problems or problems for less than a month. Overall, the total SCIQ score for the participants averages 18.1±6.2, indicating varying degrees of sleep disturbances among the study population.

**Figure 1:**
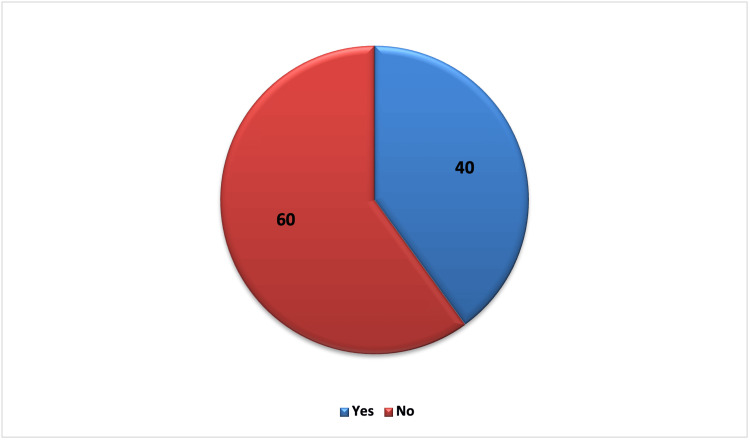
Prevalence of insomnia among participants

Figure 1 shows that 40% of the study population experiences insomnia, identified by a score of 16 or less on the eight-item SCI, which was used to assess the presence and severity of insomnia among study participants. The remaining 60% of participants scored above 16, indicating they do not meet the criteria for insomnia. The average insomnia score among participants was 18.1±6.2, indicating variability in the severity of insomnia symptoms across the population. These results highlight a significant portion of participants affected by insomnia, with varying levels of symptom severity observed.

Table 3 highlights that the DSM-5 criteria for insomnia are significantly more common among individuals with insomnia compared to those without. Among participants with insomnia, 63.9% reported difficulty initiating or maintaining sleep, compared to just 6.7% of those without insomnia, a difference that was statistically significant (p<0.001). In terms of sleep disturbance frequency, 85.4% of insomnia sufferers experienced frequent disturbances, whereas only 14.9% of those without insomnia reported such issues, and this criterion was also significant (p<0.001). Furthermore, a significant majority (89.5%) of individuals with insomnia reported significant distress due to their sleep problems, compared to 62.3% of those without insomnia, with this difference being statistically significant (p<0.001). Long-term sleep disturbances were reported by 91.3% of the insomnia group, compared to 33.1% of the non-insomnia group, and this was statistically significant (p<0.001).

**Table 3:** Association of the DSM-5 criteria for those who screen positive for insomnia versus those who do not DSM-5; Diagnostic and Statistical Manual of Mental Disorders, Fifth Edition; N: frequency; %: percentage; 1 p-value, *p<0.05, significant

Clinical criteria		Insomnia		Sig.^1^
		Yes	No	
		N (%)		
Difficulty initiating or maintaining sleep	Yes	140 (63.9%)	22 (6.7%)	<0.001*
	No	79 (36.1%)	307 (93.3%)	
Frequency of sleep disturbances	Yes	187 (85.4%)	49 (14.9%)	<0.001*
	No	32 (14.6%)	280 (85.1%)	
Significant distress	Yes	196 (89.5%)	205 (62.3%)	<0.001*
	No	23 (10.5%)	124 (37.7%)	
Duration of sleep disturbances	Yes	200 (91.3%)	109 (33.1%)	<0.001*
	No	19 (8.7%)	220 (66.9%)	

Table 4 outlines the association between various sociodemographic and clinical factors and insomnia. Among the age groups, the highest prevalence of insomnia was found in the 40-60-year group (n=91, 45.7%), followed by the 18-24-year group (n=32, 38.6%) and the 25-39-year group (n=93, 38.4%). The lowest prevalence was observed in those older than 60 years (n=3, 12.5%). The association between age and insomnia was statistically significant (p=0.014).

**Table 4:** Association of sociodemographic and clinical factors with insomnia N: frequency; %: percentage; 1 p-value, *p<0.05, significant

Factor		Insomnia		Sig.^1^
		Yes	No	
		N (%)	N (%)	
Nationality	Saudi	175 (39%)	274 (61%)	0.315
	Non-Saudi	44 (44.4%)	55 (55.6%)	
Gender	Female	198 (63.1%)	116 (36.9%)	0.094
	Male	131 (56%)	103 (44%)	
Age (years)	18-24	32 (38.6%)	51 (61.4%)	0.014*
	25-39	93 (38.4%)	149 (61.6%)	
	40-60	91 (45.7%)	108 (54.3%)	
	>60	3 (12.5%)	21 (87.5%)	
Occupation	Employed	112 (39.7%)	170 (60.3%)	0.182
	Unemployed	107 (40.2%)	159 (59.8%)	
Marital status	Single	66 (38.4%)	106 (61.6%)	0.921
	Married	144 (41%)	207 (59%)	
	Divorced	6 (37.5%)	10 (62.5%)	
	Widower	3 (33.3%)	6 (66.7%)	
Highest education level	Primary or middle school	4 (33.3%)	8 (66.7%)	0.125
	Secondary school	37 (34.3%)	71 (65.7%)	
	University	144 (44.3%)	181 (55.7%)	
	Postgraduate	34 (33.7%)	67 (66.3%)	
	Uneducated	0 (0%)	2 (100%)	
Past medical history				
Anxiety	Yes	49 (66.2%)	25 (33.8%)	<0.001*
	No	167 (35.8%)	300 (64.2%)	
Depression	Yes	27 (54%)	23 (46%)	0.033*
	No	189 (38.5%)	302 (61.5%)	
Asthma	Yes	19 (47.5%)	21 (52.5%)	0.309
	No	197 (39.3%)	304 (60.7%)	
Diabetes	Yes	13 (35.1%)	24 (64.9%)	0.538
	No	203 (40.3%)	301 (59.7%)	
High blood pressure	Yes	24 (58.5%)	17 (41.5%)	0.011*
	No	192 (38.4%)	308 (61.6%)	
Smoking status	Yes	27 (60%)	18 (40%)	0.0063*
	No	191 (38%)	312 (62%)	
Weight (kg)	<40 kg	0 (0%)	1 (100%)	0.035*
	40-49 kg	10 (28.6%)	25 (71.4%)	
	50-59 kg	31 (37.3%)	52 (62.7%)	
	60-69 kg	47 (31.8%)	101 (68.2%)	
	70-79 kg	56 (47.1%)	63 (52.9%)	
	80-89 kg	38 (47.5%)	42 (52.5%)	
	90-99 kg	17 (37.8%)	28 (62.2%)	
	>100 kg	20 (54.1%)	17 (45.9%)	
Height (cm)	140-149 cm	6 (35.3%)	11 (64.7%)	0.583
	150-159 cm	76 (38.6%)	121 (61.4%)	
	160-169 cm	77 (38.7%)	122 (61.3%)	
	170-179 cm	47 (48%)	51 (52%)	
	180-189 cm	12 (34.3%)	23 (65.7%)	
	190 cm +	1 (50%)	1 (50%)	

Among clinical factors, significant differences were noted. Anxiety was strongly associated with insomnia, with 49 individuals (66.2%) with anxiety reporting insomnia compared to 167 (35.8%) without anxiety, a statistically significant difference (p<0.001). Similarly, depression was significantly associated with insomnia, with 27 individuals (54%) with depression reporting insomnia compared to 189 (38.5%) without depression (p=0.033). High blood pressure was also significantly linked to insomnia, with 24 individuals (58.5%) with high blood pressure reporting insomnia compared to 192 (38.4%) without it (p=0.011).

Regarding smoking status, a significant association was found. Insomnia was reported by 27 smokers (60%) compared to 191 non-smokers (38%), with this difference being statistically significant (p=0.0063). In terms of weight, the highest prevalence of insomnia was among those weighing over 100 kg (20 individuals, 54.1%), followed by those in the 70-79 kg range (56 individuals, 47.1%) and the 80-89 kg range (38 individuals, 47.5%), compared to other categories (p=0.035). No other factors were found to be significantly associated with insomnia (p>0.05).

Additionally, no significant associations were observed for nationality (p=0.315), gender (p=0.094), marital status (p=0.921), occupation (p=0.182), highest education level (p=0.125), asthma (p=0.309), diabetes (p=0.538), and height categories (p=0.583).

## Discussion

Insomnia, a prevalent sleep disorder, significantly impacts individuals' physical and mental health, leading to a decreased quality of life. This study aimed to document the prevalence of insomnia and its associated risk factors among adults in Riyadh, Saudi Arabia. In our study, females constituted a significant portion (57.3%) of the sample, while males accounted for 42.7%. This gender disparity may indicate a higher willingness or availability of females to participate in such studies. The age distribution aligns with other studies showing that middle-aged individuals are often the primary workforce, reflected in their higher representation in such studies [11]. The high level of education among participants is encouraging and supports findings from other studies indicating an increasing trend in higher education attainment in the region [4,9].

In our study, the largest group of participants weighed between 60 and 69 kg, comprising 27% of the sample (148 participants). The relatively high proportion of participants in the 60-69 kg and 70-79 kg ranges reflects a common trend observed in several studies focusing on Middle Eastern populations. For example, Al-Hazzaa et al. found that the majority of young adults in Saudi Arabia fall within these weight categories, which is consistent with our results [12]. Another study by Jehan et al. found a strong association between higher body mass index (BMI) and an increased risk of insomnia due to factors such as sleep apnea and metabolic disturbances [13]. In our study, most participants fall within the normal to slightly overweight range. This implies that while extreme weight categories (underweight and obese) are not predominant in this sample, those within the normal weight range may still experience insomnia due to other factors such as stress, lifestyle, and comorbid conditions. The National Health and Nutrition Examination Survey (NHANES) also supports the association between higher BMI and sleep disturbances but notes that there is a complicated correlation between weight and insomnia impacted by various factors, including diet, physical activity, and mental health [14]. Additionally, our findings reveal that obese individuals (>100 kg) had the highest prevalence of insomnia (n=20, 54.1%, p=0.035). This suggests that among the participants, those who weighed over 100 kg had a notably higher prevalence of insomnia compared to those with lower weights. One possible explanation for this finding is the relationship between obesity and obstructive sleep apnea (OSA), a sleep disorder commonly associated with obesity, where the airway becomes blocked during sleep, leading to interruptions in breathing and poor sleep quality [13]. The frequent awakenings and disrupted sleep patterns caused by OSA can contribute significantly to insomnia [15].

The findings from the SCIQ indicate a notable prevalence of insomnia among the study participants (n=219, 40%). The total SCIQ score averaged 18.1±6.2, suggesting varying degrees of sleep disturbances. This prevalence exceeds global estimates, where insomnia affects approximately one-third of the population, with prevalence rates ranging from 10% to 33% [16,17]. The high prevalence observed in this study underlines the importance of addressing insomnia as a significant public health concern in Riyadh. Possible reasons for the high prevalence of insomnia, despite normal sleep onset latency, include stress and anxiety, which are common in modern lifestyles and can lead to sleep disturbances even if the onset of sleep is normal [18]. Medic et al. state that chronic health conditions such as hypertension, diabetes, and depression, which are prevalent in many populations, can significantly affect sleep quality and contribute to insomnia [15]. According to the National Sleep Foundation (NSF) guidelines, normal sleep onset latency is considered to be less than 30 minutes [19]. Our findings, where the majority fall within this time range (n=285, 52%), align with these guidelines. However, the subset of participants taking more than 61 minutes to fall asleep is concerning and highlights the presence of significant sleep onset insomnia (n=36, 6.6%). Additionally, we found that insomnia was significantly associated with the DSM-5 criteria, including difficulty initiating or maintaining sleep, frequency of sleep disturbances, significant distress, and duration of sleep disturbances (p<0.001 for each). This is consistent with another study by Shi et al., which found that prolonged sleep onset latency and nighttime awakenings are common in individuals with chronic insomnia [20].

In the present study, sleep quality was rated as very good by 28.8% of participants, good by 26.3%, and average by 32.1%. A study by Simonelli et al. reported that about one-third of adults rate their sleep quality as poor or very poor, which is comparable to the 12.8% of our participants who reported poor or very poor sleep quality [21]. Another survey by Fabbri et al. indicated that poor sleep quality affects 30% of adults globally, which aligns closely with the 32.1% of our participants reporting average sleep quality [22]. Our finding that 38.9% of participants reported a moderate to severe impact on various aspects, such as mood, energy, and interpersonal relationships, is consistent with the results of a study by Konjarski et al., which found that poor sleep significantly impacts mood, energy, and interpersonal relationships [23].

The present study found no significant difference between Saudi and non-Saudi participants regarding the presence of insomnia (p=0.315). This suggests that nationality does not play a significant role in insomnia prevalence among the participants. Similar findings were reported in a study by Hammoudeh et al., in the Gulf region, which found no significant differences in sleep disorders across different nationalities [24]. There were significant differences across age groups regarding the presence of insomnia (p=0.014). Participants aged 40-60 had the highest prevalence of insomnia (n=91, 45.7%), indicating poorer sleep quality in older populations. This finding contrasts with other studies, like Ohayon and Reynolds, which reported increased insomnia prevalence with age due to factors such as health problems, medication use, and changes in sleep architecture [25]. Marital status did not significantly affect insomnia prevalence, with singles, married individuals, divorced individuals, and widowers showing similar prevalence (p=0.921). This contrasts with findings from the study by Chen et al., which suggested that married individuals generally report better sleep quality due to social support [26]. However, the current study's results indicate that marital status alone may not be a strong predictor of insomnia. Participants with anxiety (n=49, 66.2%, p<0.001) and depression (n=27, 54%, p=0.033) had a significantly higher prevalence of insomnia compared to those without these conditions, indicating worse sleep quality. This aligns with some studies that highlight the strong association between mental health disorders and insomnia [15,17]. High blood pressure was also significantly associated with a high prevalence of insomnia (n=24, 58.5%, p=0.011). This is consistent with studies linking chronic health conditions to sleep disturbances [16]. Smokers had significantly higher insomnia rates compared to non-smokers (n=27, 60%, p=0.0063), reflecting the negative impact of smoking on sleep quality. This finding aligns with the study by Jaehne et al., which reported that nicotine dependence is associated with increased sleep disturbances [27].

In addition, the prevalence of insomnia did not significantly differ based on educational levels (p=0.125). This finding contradicts another study which reported that sleep difficulties are more common in those with lower levels of education, potentially due to socioeconomic stressors and lower health literacy [28].

Limitations

This study has several limitations that should be acknowledged. First, the cross-sectional design limits the ability to determine causal relationships between sociodemographic variables and insomnia. Second, the reliance on self-reported data may introduce recall bias and affect the accuracy of the results. Additionally, the research did not consider other possible confounding factors, such as lifestyle factors, dietary habits, and genetic predispositions, which could influence the prevalence of insomnia.

## Conclusions

This study highlights the significant prevalence of insomnia among adults in Riyadh, Saudi Arabia, and identifies various sociodemographic factors that influence sleep quality. Poor sleep quality was particularly observed among older adults, smokers, and obese individuals. Additionally, those with chronic health conditions such as anxiety, depression, and high blood pressure had higher rates of insomnia. The findings underscore the need for targeted public health interventions to address insomnia and its associated risk factors. Future research should focus on longitudinal studies to establish causal relationships and explore the impact of lifestyle and genetic factors on insomnia. Furthermore, increasing the study's sample size to be more representative and varied across Saudi Arabia's regions would enhance the generalizability of the results and help shape more effective health policies and initiatives.
